# Dysregulation of Cytokine Response in Canadian First Nations Communities: Is There an Association with Persistent Organic Pollutant Levels?

**DOI:** 10.1371/journal.pone.0039931

**Published:** 2012-07-02

**Authors:** Pascal Imbeault, C. Scott Findlay, Michael A. Robidoux, François Haman, Jules M. Blais, Angelo Tremblay, Susan Springthorpe, Shinjini Pal, Tim Seabert, Eva M. Krümmel, Rasha Maal-Bared, Jason A. Tetro, Sunita Pandey, Syed A. Sattar, Lionel G. Filion

**Affiliations:** 1 Behavioral and Metabolic Research Unit, School of Human Kinetics, Faculty of Health Sciences, University of Ottawa, Ottawa, Ontario, Canada; 2 Indigenous Health Group, School of Human Kinetics, Faculty of Health Sciences, University of Ottawa, Ottawa, Ontario, Canada; 3 Department of Biology and Institute of Environment, University of Ottawa, Ottawa, Ontario, Canada; 4 Division of Kinesiology, Faculty of Medicine, Laval University, Québec, Québec, Canada; 5 Centre for Research on Environmental Microbiology, University of Ottawa, Ottawa, Ontario, Canada; 6 Department of Biochemistry, Microbiology and Immunology, Faculty of Medicine, University of Ottawa, Ontario, Canada; 7 Centre for Cancer Therapeutics, Ottawa Hospital Research Institute, Ottawa, Ontario, Canada; Maastricht University Medical Center, Netherlands

## Abstract

In vitro and animal studies report that some persistent organic pollutants (POPs) trigger the secretion of proinflammatory cytokines. Whether POP exposure is associated with a dysregulation of cytokine response remains to be investigated in humans. We studied the strength of association between plasma POP levels and circulating cytokines as immune activation markers. Plasma levels of fourteen POPs and thirteen cytokines were measured in 39 Caucasians from a comparator sample in Québec City (Canada) and 72 First Nations individuals from two northern communities of Ontario (Canada). Caucasians showed significantly higher levels of organochlorine insecticides (β-HCH, p,p′-DDE and HCB) compared to First Nations. Conversely, First Nations showed higher levels of Mirex, Aroclor 1260, PCB 153, PCB 170, PCB 180 and PCB 187 compared to Caucasians. While there was no difference in cytokine levels of IL-4, IL-6, IL-10 and IL-22 between groups, First Nations had significantly greater average levels of IFNγ, IL-1β, IL-2, IL-5, IL-8, IL-12p70, IL-17A, TNFα and TNFβ levels compared to Caucasians. Among candidate predictor variables (age, body mass index, insulin resistance and POP levels), high levels of PCBs were the only predictor accounting for a small but significant effect of observed variance (∼7%) in cytokine levels. Overall, a weak but significant association is detected between persistent organochlorine pollutant exposure and elevated cytokine levels. This finding augments the already existing information that environmental pollution is related to inflammation, a common feature of several metabolic disorders that are known to be especially prevalent in Canada's remote First Nations communities.

## Introduction

Persistent organic pollutants (POPs) are a wide range of compounds that include dioxins, polychlorinated biphenyls, organochlorine pesticides and perfluorinated acids. These partially volatile compounds, largely of industrial origin, can be transported large distances from their source via global distillation [1]. Owing to both persistence and lipophilicity, POPs have comparatively high bioaccumulative potential [2]. Global legislation [3] has led to a decline in human exposure to most legacy POPs over the last three decades, even in remote communities [4]. However, due to the global flow of POPs in the environment [5], [6], remote areas, especially arctic and subartic regions, continue to be at risk of sustained contamination [7], [8]. Many individuals living in these regions, including First Nations and Inuit communities, show elevated POP exposure, as suggested by their higher levels of POPs in blood [9] and adipose tissues [10] as compared to individuals living in a southern region of Canada.

There is, moreover, accumulating evidence of potential impacts of POPs exposure on human health that might be mediated by a variety of mechanisms, including components of the human immune system. There is epidemiological evidence spanning a range of study populations and experimental designs that suggest a diabetogenic effect of POPs exposure (reviewed in [11]). The association between elevated POPs exposure and diabetes has been documented in specific populations exposed occupationally, recreationally or through specific industrial accidents [12]–[17], or more recently, from samples, that are, at least notionally, more representative of the general population [18]. POPs exposure has also been consistently associated, in a dose-dependent manner, with increased risk of ischemic heart disease mortality [19]. A weaker association between POPs exposure and risk of all cardiovascular diseases has also been described. Finally, several cross-sectional and prospective studies have also reported positive associations between plasma PCB levels and excess adipose tissue mass, as estimated by body mass index [20]–[24].

In Canadian First Nations communities, adult overweight and obesity prevalences (∼73%) have been shown to be disproportionally higher compared to the general Canadian population (∼50%) [25]. This high prevalence of obesity among First Nations people was also identified to be partly responsible for the greater prevalence of self-reported diabetes in this population (3 to 5 times higher than the general Canadian population) [26]. A low degree of physical activity as well as poor food availability and dietary choices are key factors well recognized to contribute to the high prevalence of metabolic disorders observed in First Nations individuals [27]. Despite this rapid (within 50 years) and profound shift in lifestyle and dietary habits, locally harvested and prepared foods are still of tremendous value for First Nations people [28], [29]. However, as we recently reported, the reliance on traditional foods in remote First Nations communities of Northern Ontario may increase contaminant exposure. Indeed, we found a strong association between the frequencies of wild food consumption and plasma POP concentrations (unpublished work). Whether elevated POP exposure is associated with the activation of the immune response remains unknown. In this study, we investigate this association in a sample composed of Caucasians living in the southern part of the province of Québec (Canada) and First Nations (Oji-Cree) adults living in remote communities from northwestern Ontario (Canada).

## Methods

All research activities underwent ethics review and were approved by the research ethics boards of the University of Ottawa, Laval University and Health Canada. Written consent was obtained from all participants involved in our study.

### Participants

#### Caucasians

Thirty nine individuals from Québec City (Canada) were recruited through the media and gave their written consent to participate in a study originally designed to induce weight loss, as previously reported [30]. All individuals underwent a medical evaluation by a physician, which included a medical history. No participant was pregnant and none was type 1 diabetic. None was working in an environment where the risk of exposure to POPs was high. None had recently been on a diet or involved in a weight-reduction program, and for all participants, body weight had been stable for all participants during the six months prior to the study.

#### First Nations

Seventy-two people over 19 years of age were recruited from two communities in northwestern Ontario (Canada). The populations of these two communities were 372 and 164 people, respectively. Participants were recruited and interviewed through the assistance of local research coordinators and translators who were hired over the course of the study, as recently described [31]. Overall, the participating individuals represent 24% and 9%, respectively, of the eligible adult population of the two communities studied. Participants were recruited based on self-described dietary behaviour as either relying predominantly on land-based food items (primarily wild game) or predominantly store-bought foods. To be included in the study, a candidate had to be Aboriginal, over 18 years of age, not pregnant and free of type 1 diabetes. None had recently been on a diet or involved in a weight-reduction program and their body weight had been stable during the six months prior to the study. Dietary behaviour of participants was determined from semi-structured interviews and participation/direct observation in daily food practices. No community members relied exclusively on either wild food or store-bought foods. Rather, there was a continuum of wild food consumption ranging from eating land based foods every day to eating less than once a month, as previously documented [29].

### Anthropometric and insulin sensitivity assessments

Caucasians and First Nations participants were asked to report to the laboratory or the local community nursing clinic, respectively, in the morning following 24 h without heavy physical activity and after a 9 h fast.

Body weight was determined with a standard beam scale and height and waist circumference were measured with a measuring tape. Height was measured with the participant's bare feet together, with heels, back, and head against the wall, and following a normal inspiration. Waist circumference was measured, in duplicate (and averaged) at the mid-point between the last floating rib and the top of the iliac crest [32].

### Insulin and glucose measurements

Participants were asked to fast for 9 h and to refrain from smoking, exercising and drinking (except water) preceding the blood draws. If they had taken any medication in nine hours preceding the blood draws, this was also noted. Upon collection, samples were immediately centrifuged at 3500 rpm and plasma was temporarily stored at −20°C in the clinics before being transferred to the laboratory where they were stored at −80°C for future assays.

Glucose concentrations were assayed using spectrophotometric analysis (340 nm) after conversion of glucose to glucose 6-phosphate by hexokinase and insulin levels were analyzed by a commercially available enzyme-linked immunosorbent assay (ELISA) kit (Millipore, Billerica, MA, USA), as previously described [33]. Insulin resistance was quantified using Homeostasis Model Assessment (HOMA-IR) [34]. In summary, the HOMA-IR values were established by dividing the product of the fasting plasma glucose (FPG; mmol/L) and insulin (FPI; µIU/mL) values by 22.5.

### Contaminant analysis

All POP measurements were conducted by the Toxicology Centre at the National Institute of Public Health of Quebec. Each sample was assayed for the following contaminants: Aroclor 1260, polychlorinated biphenyl (PCB)28, PCB52, PCB99, PCB101, PCB105, PCB118, PCB128, PCB138, PCB153, PCB156, PCB163, PCB170, PCB180, PCB183, PCB187, aldrin, α-chlordane, γ-chlordane, β-(hexachlorocyclohexane) HCH, *cis*-nonachlor, *trans*-nonachlor, 2,2′-bis(4-chlorophenyl)-1,1-dichloroethene (DDE), 2,2′-bis(4-chlorophenyl)-1,1-trichloroethene (DDT), hexachlorobenzene (HCB), mirex, oxychlordane. Plasma samples were enriched with internal standards and denatured with formic acid. The compounds were then extracted from the aqueous matrix using solid phase separation and extracts were cleaned using florisil columns prior to analysis. Plasma samples were eluted from columns using methylene chloride-hexane (25∶75 vol/vol) and analyzed on an E-446 Agilent 6890 GC-MS (gas chromatographer- mass spectrometer) equipped with dual capillary columns. The concentration of each analyte was determined using percent recovery of labeled internal standards, as previously described [35]. The ECD (electron capture detector; Agilent G2397A) served to verify the detection limits for PCB congeners 28 and 52.

Mercury levels in hair were analyzed at the Laboratory for the Analysis of Natural and Synthetic Environmental Toxins (LANSET) at the University of Ottawa. Hair was clipped as close as possible to the scalp and cleaned in a 2∶1 chloroform: methanol solution once transported back to the laboratory. Total mercury levels were isolated using a mercury SP-3D analyzer (Nippon Instruments Corporation, Japan) and detected using cold vapour atomic absorption spectroscopy.

A particular POP was not evaluated during analysis if more than 40% of individuals from the entire sample had values below the instrument detection limit (IDL). These included: aldrin, α-chlordane, γ-chlordane, cis-nonachlor, DDT, PCBs 28, 52, 99, 101, 105, 128, 163 and 183. For 7 of the 14 POPs considered for the analyses, between 2 and 22 participants had values below the IDL. Half the IDL was given to participants who had a specific POP value below the IDL. The other 7 POPs (which included DDE, oxychlordane, Aroclor1260, PCBs 138, 153, 180 and 187) had a 100% detection frequency.

### Cytokine measurements

Cytokine levels were measured in each sample using Flowcytomix human Th1/Th2 11plex kit, interleukin (IL)17A and IL-22 simplex kits (Bender Medsystems GmbH, Austria) as per the manufacturer's instructions. The detection limit for interferon (IFN)γ, IL-17A, IL-22, IL-8 IL-6, TNFα, IL-1β, IL-4, IL-5, IL-10, IL-2, IL-12p70 and TNFβ were 1.6, 2.5, 43.3, 0.5, 1.2, 3.2, 4.2, 20.8, 1.6, 1.9, 1.5 and 2.4 pg/ml, respectively. The data were acquired as outlined by Bender Flow systems on a FC500 MPL flow cytometer (Beckman Coulter, Toronto ON) and the data were analyzed using FlowCytomix™ Pro 2.3 software. The standards used for determining cytokine levels were measured in duplicate.

### Statistical analysis

Data in the text and tables are expressed as the mean ± standard deviation while data in figures are expressed as mean ± standard error of the mean. All statistical analyses were performed using SPSS for Windows Version 17.0 (SPSS Inc., Chicago, USA). A level of significance of p<0.05 was considered statistically significant. The chi-square (χ^2^) test of association was used to determine whether the prevalence of diabetes was independent between groups. Variables not normally distributed were transformed mathematically (i.e. logarithmic transformation) before analyses. The Student's t test was utilized for comparisons between Caucasians and First Nations individuals. POPs and cytokine levels were compared between groups after adjusting for age and/or obesity levels. Principal component analyses (PCA) based on the correlation matrix were used to identify composite contaminant and cytokine variables. Associations between candidate predictors (age, body mass index, insulin resistance, first and second components extracted from the pooled contaminant matrix) and the levels of composite cytokine variables derived from PCA was evaluated by fitting multiple regression models.

## Results

### Descriptive characteristics

The sample included 39 Caucasians from Québec City and 72 Northern Ontario First Nations (FN) individuals (Table 1). Overall, men made up 44% of all participants (Caucasians: 18 men and 21 women; First Nations: 31 men and 41 women). The prevalence of type 2 diabetes was significantly greater in First Nations than in Caucasians participants (χ^2^ = 19.7, df = 1, p<0.0001). Mean age of both groups was comparable (t(109) = −0.29, not significant (NS)). However, average weight (t(109) = −2.50, p<0.01) and BMI (t(109) = −3.63, p<0.001) were significantly greater in Caucasians than in First Nations individuals. Average waist circumference was significantly higher in First Nations as compared to Caucasians (t (106) = 3.67, p<0.001). Fasting glucose levels were significantly greater in First Nations than in Caucasians individuals (t (109) = 3.80, p<0.001) while HOMA-IR, a surrogate of insulin resistance, did not differ between groups (t (109) = 0.37, NS).

**Table 1:** Characteristics of participants.

	Caucasians (n = 39)	First Nations (n = 72)
Sex (Men/Women)	18/21	31/41
Diabetes (yes/no)	1/38	26/46 *
Age (year)	43±5	46±15
Weight (kg)	97±14	90±17 *
BMI (kg/m^2^)	35.2±3.5	32.3±4.4 *
Waist circumference (cm)	104±12	115±11 *
Fasting glucose (mmol/l)	5.2±0.9	6.8±2.6 *

Data are expressed as means ± standard deviation.

BMI, body mass index.

Significant difference between groups at.

* p<0.05.

### Persistent organic pollutant (POP) levels

**Figure 1:**
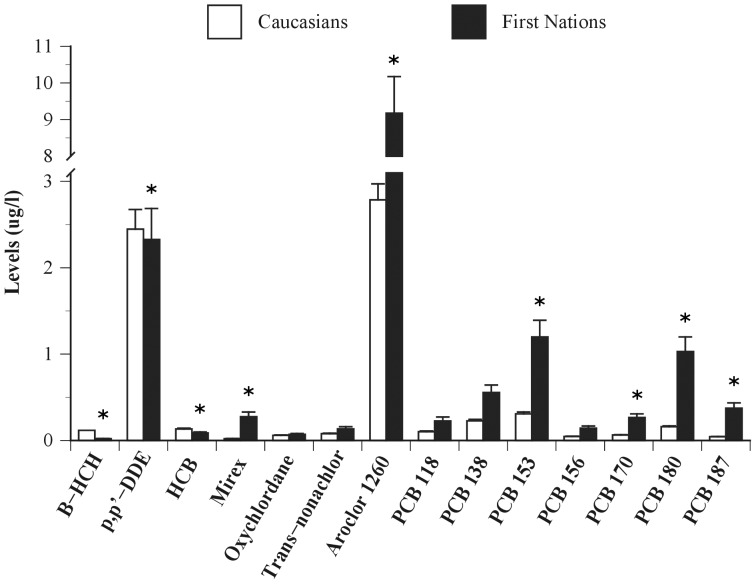
Plasma persistent organic pollutant levels (in µg/l) of Caucasians from the southern province of Québec (Canada) and First Nations from northern remote communities of Ontario (Canada). Significant difference between groups at * p<0.05.

Caucasians showed significantly greater average levels of β-HCH (t(109) = −12.82, p<0.0001), p,p′-DDE (t(109) = −2.26, p<0.05) and HCB (t(109) = −4.88, p<0.0001) compared to First Nations people (Figure 1). Conversely, First Nations individuals showed greater levels of Mirex (t(109) = 6.00, p<0.0001), Aroclor 1260 (t(109) = 2.38, p<0.05), PCB 153 (t(109) = 3.03, p<0.01), PCB 170 (t(109) = 2.65, p<0.01), PCB 180 (t(109) = 4.78, p<0.0001) and PCB 187 (t(109) = −5.29, p<0.0001) compared to Caucasians. No statistical differences among groups were detected for Oxychlordane (t(109) = −1.51, NS), Trans-nonachlor (t(109) = −0.51, NS), PCB 118 (t(109) = −0.32, NS), PCB 138 (t(109) = 1.19, NS) and PCB 156 (t(109) = 0.16, NS).

Principal component (PC) analysis of plasma POP concentrations extracted for each group separately showed a high degree of similarity in the pattern of associations among groups. Component extraction based on the pooled sample revealed two major components accounting for 76% and 15% of the total variance in contaminant levels (Table 2). PC 1, for which all factors loaded highly and positively, is interpreted as a generalized index of POP burden. The second principal component contrasted β-HCH, p,p′-DDE, HCB, Oxychlordane, trans-Nonachlor and PCB 118 (mainly insecticides) (positive loadings) on the one hand, and Mirex, Aroclor 1260, PCBs 153, 170, 180 and 187 (mainly PCBs) on the other hand. Comparison of PC scores showed no difference in PC 1 between FN and Caucasians (t(109) = 1.2, NS) but detectable differences in mean PC 2, with Caucasians exhibiting higher pesticides and lower PCB levels than First Nations individuals (t(109) = −16.35, p<0.0001) even when corrected for body weight. Comparable results were observed when principal component analyses were performed on POP levels expressed per lipid basis (i.e. µg/kg lipids) (results not shown).

**Table 2:** Eigenvectors from principal component analysis of plasma persistent organic pollutant levels of pooled Caucasians and First Nations individuals.

	Pooled samples	
	Components	
	1	2
ß-HCH	0.39	0.50
p,p′-DDE	0.68	0.46
HCB	0.21	0.39
Mirex	0.26	−0.31
Oxychlordane	0.28	0.23
Trans-nonachlor	0.35	0.84
Aroclor 1260	0.30	−0.08
PCB 118	0.28	0.13
PCB 138	0.30	0.004
PCB 153	0.30	−0.12
PCB 156	0.28	−0.01
PCB 170	0.29	−0.17
PCB 180	0.28	−0.24
PCB 187	0.27	−0.28
Eigenvalue	10.70	2.13
Percent	76.4	15.2

### Cytokine levels

**Figure 2:**
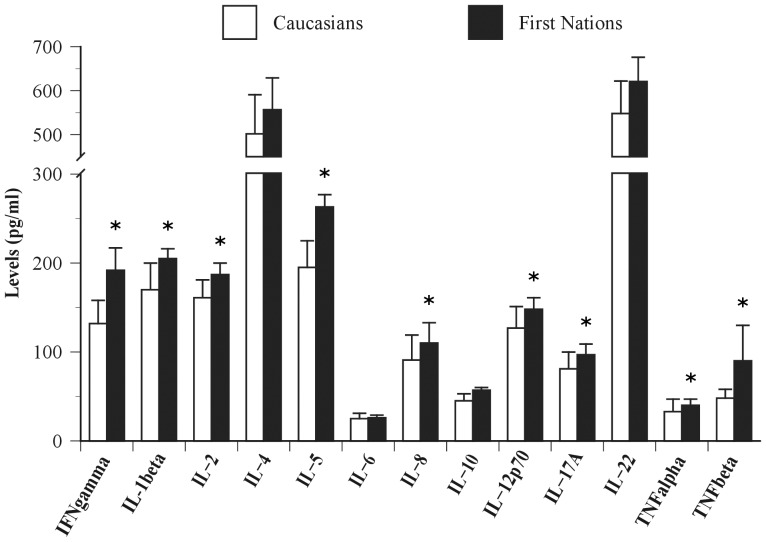
Plasma cytokine levels of Caucasians from the southern province of Québec (Canada) and First Nations from northern remote communities of Ontario (Canada). Significant difference between groups at * p<0.05.

First Nations individuals showed significantly greater average levels of IFNγ (t(109) = 2.91, p<0.01), IL-1β (t(109) = 2.46, p<0.05), IL-2 (t(109) = 2.39, p<0.05), IL-5 (t(109) = 2.55, p<0.01), IL-8 (t(109) = 2.15, p<0.05), IL-12p70 (t(109) = 1.96, p<0.05), IL-17A (t(109) = 2.09, p<0.05), TNFα (t(109) = 3.60, p<0.001) and TNFβ (t(109) = 2.42, p<0.05) compared to Caucasians (Figure 2). No statistical differences between groups were detected for levels of IL-4 (t(109) = 1.78, NS), IL-6 (t(109) = 1.89, NS), IL-10 (t(109) = 1.86, NS) and IL-22 (t(109) = 1.69, NS).

Principal component analysis of all immunological parameters showed similar associations in each group. Component extraction based on the pooled sample identified two components accounting for 81% and 6% of the total variance (Table 3). PC1 is a generalized immunological profile index with all variables showing high positive loadings. PC2 was a contrast between IL-5, IL-6, IL-8, IL-22 and TNFβ (positive loadings) on one hand and IFNγ, IL-4, IL-12p70, IL-17A and TNFα on the other hand. Comparison of PC scores showed greater average levels of PC1 in FN individuals compared to Caucasians (t(109) = 2.62, p<0.01), a difference which persisted even when corrected for body weight. No statistical difference between groups was detected for PC2 (t(109) = −1.05, NS).

**Table 3:** Eigenvectors from principal component analysis of plasma cytokine levels of pooled Caucasians and First Nations individuals.

	Pooled samples	
	Components	
	1	2
IFNγ	0.29	−0.14
IL-1β	0.30	0.06
IL-2	0.29	−0.07
IL-4	0.29	−0.14
IL-5	0.30	0.10
IL-6	0.29	0.14
IL-8	0.24	0.54
IL-10	0.29	−0.002
IL-12p70	0.28	−0.17
IL-17A	0.28	−0.28
IL-22	0.25	0.46
TNFα	0.22	−0.56
TNFβ	0.29	0.07
Eigenvalue	10.58	0.78
Percent	81.4	6.0

### Predicting cytokine levels

We regressed the first cytokine principle component (PC1 (cytokines)), interpreted as a general index of cytokine levels, on the first and second components extracted from the pooled contaminant sample, age, BMI and HOMA levels. The full model had low predictive power, accounting for less than 10% of the total variance in PC1 (cytokines), with only (POPs) making a statistically significant contribution to model fit (t = −2.80, df = 105, p = 0.006; partial r^2^ = 0.067).

Using the First Nations sample, we also regressed cytokine levels on the first two components of the POP matrix, age, body mass index, HOMA levels and community as a categorical predictor. The full model accounted for 18% of observed variance in PC 1 (cytokines) levels with age (t = 2.09, df = 65, p = 0.04; partial r^2^ = 0.055) and community (community 1) (t = −2.59, df = 65, p = 0.01; partial r^2^ = 0.085) showing significantly statistical detectable contributions to model fit.

### Mercury

There was no difference observed in average hair mercury concentrations between the two First Nations communities (Community 1: 1923±1807 (mean ± SD); Community 2: 1726±1367 ppb, NS). When included in the predictive model for cytokines described above, hair mercury levels made a negligible contribution to overall model fit.

## Discussion

The aim of this study was to determine the strength of association between plasma levels of persistent organic pollutants (POPs) and a specific component of the immune system, as defined by circulating cytokines, in humans. The study sample was composed of Caucasians from a southern urban center (Québec City, Canada) and First Nations adults living in two remote northwestern communities of Ontario (Canada). Mean average plasma levels of a number of specific PCB are ∼2 to 7 times greater in First Nations people than in Caucasians. Conversely, levels of organochlorine insecticides are ∼1.2 to 13 times higher in Caucasians compared to First Nations individuals. Our results also indicate significantly greater average levels of many cytokines (i.e. INFγ, IL-1β, IL-2, IL-5, IL-8, IL-12p70, IL-17A, TNFα and TNFβ) in First Nations individuals compared to Caucasians. Finally, this study shows that high levels of PCBs are the only predictor accounting for a small but significant effect of observed variance (e.g. ∼7%) in cytokine levels.

POPs include chemicals such as polychlorinated biphenyls, organochlorine insecticides, polychlorinated dioxins and furans, perfluorinated acids, and brominated flame retardant. In this study, we report that First Nations individuals from a remote northwestern communities in Ontario have significantly higher (∼2 to 7 fold) levels of PCBs but significantly lower (∼1.2 to 13 fold) levels of organochlorine insecticides than those of Caucasians from a southern urban centre in the province of Québec. Owing to their hydrophobicity and their resistance to degradation by light, chemical or biological processes, POPs bioaccumulate sharply in lipids [36]. POPs can therefore bioaccumulate through food absorption such that the greatest concentrations are found in species that occupy the highest positions in food webs. The traditional dietary habits of First Nations people studied here likely explain their higher plasma levels of PCBs as compared to those in Caucasians. Indeed, using a mixed-method ethnological approach, we documented that First Nations individuals consume a diverse range of locally-harvested wild foods such as fish and game [29]. We recently reported that these locally-harvested wild foods contained high levels of PCBs exceeding the consumption guidelines outlined by the United States Environmental Protection Agency (unpublished work). As mentioned above, POPs are also volatile and thus capable of long-range atmospheric transport. The progressive migration of semi-volatile POPs towards colder northern climates has also been found to contribute to the enrichment of POPs in northern latitudes [37]. As such, the geographical location (i.e. Boreal environment of northern Ontario) of the First Nations individuals studied here is another potential determinant of their higher plasma levels in PCBs as compared to those in Caucasians. The significantly lower levels of organochlorine insecticides (namely HCB, p,p′-DDE and β-HCH) found in First Nations individuals are not as easily explained. This difference may come from the fact that First Nations individuals studied here live in a remote rural area and are likely less exposed to organochlorine insecticides through old landfills, storage, waste and contaminated soils than participating Caucasians who live in an urban center.

In the current literature, the data on the effects of POPs on cytokine levels are incomplete at best and a thorough examination of various components of the immune system in experimental trials involving humans is not yet available. One of the best surrogate markers of the immune system are cytokines that are used to define specific components of the immune system, i.e. Th1 cytokines: INFγ, IL-2, IL-12; Th2: IL-4, IL-5; Treg cytokines: IL-10; Th17 cytokines: IL-17A, IL-22; pro-inflammatory cytokines: IL-1β, IL-6, TNFα, TNFβ and the neutrophil-recruiting cytokine: IL-8. Cytokines are usually short-lived molecules that exert their effects on immune and non-immune cells. Sustained cytokine overproduction could lead to alterations in metabolism [38]. In this regard, higher levels of TNFα and IL-6 have been associated with excess adiposity levels [39]–[42] and insulin resistance [39], [41], [42]. Additionally, it is now recognized that IL-1β, for which levels were significantly higher in our First Nations sample, is implicated in the disruption of insulin signaling [43] and the severity of type 2 diabetes [44]. Novel research has also demonstrated the involvement of IL-1β, IL-2, IL-8, and TNFα dysfunction in neurological diseases including attention-deficit hyperactivity disorder (ADHD) [45] and delayed cognitive development [46]. To our knowledge, the present study is the first to document an increase in immune activation as measured by a complete cytokine profile of individuals that have been characterized by a wide spectrum of POP levels. We report that the average levels of most of the cytokines measured in the current study are higher in First Nations peoples than in Caucasians and that both populations have elevated cytokine levels compared to healthy controls from a standard laboratory cohort [47].

We found that the second POPs principal component, interpreted as high levels of PCBs on the one hand and low levels of organochlorine insecticides on the other hand, accounted for ∼7% of the variance of the general index of cytokine levels (PC1 cytokine) in the sample studied. These results are consistent with the finding that PCBs may compromise the normal function of some cells such as vascular endothelial [48] or adipose cells [49] and then trigger proinflammatory events. We are, however, aware that even though we took into account key determinants known to modulate cytokine levels such as age, obesity levels and insulin resistance, factors other than PCBs, not measured in the current study, could contribute to the greater levels of cytokines observed in participating First Nations individuals. Other factors that may contribute to elevated cytokine levels may be the presence of other diseases such as arthritis and cardiovascular and chronic respiratory diseases experienced by First Nations people [50].

From field research in both First Nations communities, it was observed that landfill sites, in which common disposal practices involve burning waste, are adjacent to communities within close proximity to critical waterways. These sites, in addition to the long range transport of POPs, constitute potential sources of contaminants in Boreal communities. When comparing the two First Nations communities, no statistical difference in plasma POP levels was observed. Cytokine levels were, however, significantly greater in one community (Community 2) than the other (results not shown). Whether this difference in cytokine levels is associated with metabolic disorders and/or other contaminants not measured in this study remains to be explored.

Some limitations and strengths of this study warrant discussion. First, our limited geographical representation of the First Nations populations precludes extrapolation of our results to other First Nations communities. Another potential limitation lies in how cytokine levels can be interpreted. Cytokines are produced by a variety of cells and their levels can be acutely modulated by several factors. In the current studies, despite blood being drawn in similar clinical settings and at the same time of day, only one blood sample was taken per individual and thus results should be interpreted with caution. Plasma samples from our comparator group were obtained from a study performed more than 10 years ago from which a disclosure for future work was included in the original letter of consent. It also needs to be noted that our comparator group was selected not as representative of the Canadian population at large, but rather to match reported adiposity levels of our First Nations sample. Interestingly, despite the fact that the Caucasian group had greater weight and body mass index than the First Nations group, their average contaminant and cytokine levels were significantly lower than those of First Nations. Given that serum organochlorine concentrations are generally positively associated with body fat mass, it is quite likely that a comparator sample more representative of the Caucasian population (i.e. lower BMI) would have resulted in even greater observed differences in contaminant levels between Caucasians and First Nations groups. The results of this study are strengthened by the fact that anthropometric measurements as well as diabetes status, both of which affect cytokine levels, were objectively taken into account rather than being self-reported. Another strength of our study was the use of principal components to interpret cytokines and POPs, rather than examining individual values in multiple comparisons.

In conclusion, the present study confirms that individuals from remote northwestern Ontario First Nations communities are exposed to higher levels of persistent organic pollutants, namely PCBs, than Caucasians from the southern Canada comparator group. Our results also highlight that cytokine levels are significantly elevated in First Nations people compared to Caucasians and that a small, but significant, part of this immune activation is explained by PCBs burden. Immunity and metabolism are intimately linked and a dysfunction of this interface can lead to various metabolic disorders. This reinforces the hypothesis that POPs exposure, through its effect on the immune system, may contribute to the high rate of metabolic disorders and community health effects in different segments of the world's populations, most specifically in Canada's remote First Nations communities.
